# Complete Genome Sequence of *Bacillus* sp. Strain NC3, Isolated from *Trichonephila* Spider Ground Extract

**DOI:** 10.1128/mra.01110-21

**Published:** 2022-02-10

**Authors:** Masahiro Ito, Satomu Hasunuma

**Affiliations:** a Faculty of Life Sciences, Toyo University, Itakura-machi, Gunma, Japan; b Bio-Resilience Research Project, Toyo University, Itakura-machi, Gunma, Japan; c Bio-Nano Electronics Research Centre, Toyo University, Kawagoe, Saitama, Japan; University of Maryland School of Medicine

## Abstract

*Bacillus* sp. strain NC3, an aerobic Gram-positive bacterium, was isolated from a suspension of ground *Trichonephila clavata* specimens in saline in Japan. Here, we report the complete genome sequence of this bacterium, which has a 3.72-Mbp genome, containing 3,717 protein-coding sequences, 24 rRNA-coding sequences, and 81 tRNA-coding sequences.

## ANNOUNCEMENT

We report the whole-genome sequence of *Bacillus* sp. strain NC3, which was isolated from ground *Trichonephila clavata* specimens in Japan (36.2231N, 139.6347E). The collected spiders were ground using a Beads Crusher μT-01 (TAITEC, Japan). The ground sample was suspended in sterile saline and spread onto modified neutral complex medium (15.5 g of K_2_HPO_4_, 4.5 g of KH_2_PO_4_, 0.05 g of MgSO_4_·7H_2_O, 0.34 g of citric acid, 0.5 g of peptone, 0.2 g of yeast extract, 5 g of glucose, and 11.7 g of NaCl per liter of deionized water [pH 7.0]) plates containing 400 mM cesium chloride (pH 7.0). Strain NC3 was isolated in this way. This bacterium appeared to be most closely related to Bacillus altitudinis 41KF2b^T^, based on 16S rRNA gene sequence identity (1).

A single colony of NC3 was grown in modified neutral complex medium at 30°C for 18 h (2), and genomic DNA was prepared using a Genomic-tip 20/G (Qiagen, Japan) according to the manufacturer’s instructions. For long-read sequencing, a DNA library with barcodes added to a sample using a native barcoding expansion kit (Oxford Nanopore Technologies [ONT], UK) was prepared using a ligation sequencing kit and sequenced with a GridION sequencer (ONT) on an R9.4.1 flow cell. Raw sequence data were base called using Guppy (v4.0.11+f1071ce) (3). The adapter sequences were removed using Porechop (v0.2.3) (4) and reads of 1,000 bases or less were removed using Filtlong (v0.2.0) (5), yielding 220,304 high-quality paired-end reads. The average length of the long reads was 9,460.5 bp, and the total number of base pairs was 2,318,728,121. For short-read sequencing, a DNA library was prepared using the MGIEasy FS DNA library preparation set (MGI Tech, China) according to the manufacturer’s instructions, and the library's quality was confirmed using a fragment analyzer and double-stranded DNA (dsDNA) 915 reagent kit (Advanced Analytical Technologies Inc., USA). The reaction time for enzyme cleavage was 4 min, and fragments with an average length of 442 bp were prepared. Circularized DNA was prepared using the prepared library and the MGIEasy circularization kit according to the manual. DNBSEQ 2 × 200-bp paired-end sequencing was performed using a DNBSEQ-G400 sequencing instrument according to the manufacturer’s instructions. The adapter sequences were removed using Cutadapt (v2.7) (6). About 3.5 million read pairs (1.05 Gbp) were sampled from the sequence, from which the adapter sequences were removed using Seqkit (v0.11.0) (7); Sickle (v1.33) (8) was used to remove bases with quality scores of less than 20, and reads with a base number of fewer than 127 bases and their paired reads were discarded, yielding 6,204,960 high-quality paired-end reads. The average length of the short reads was 200 bp, and the total number of base pairs was 2,469,058,400. The *N*_50_ value was 3,715,529 bp.

High-quality short-read and long-read sequence data were assembled under the default conditions of Unicycler (v0.4.7) (9), and the genome was circularized. The results of the coding graph assembled using Bandage (v0.8.1) (10) were confirmed, and the integrity of the assembled genomic data was confirmed using CheckM (v1.1.2) (11). The coverage calculated from the total bases used for assembly was 1,289×. Default parameters were used for all software except where otherwise noted. The final chromosome sequence was 3,715,529 bp (G+C content, 41.4%). Automatic annotation was performed using Annotated at DFAST (12), which predicted 3,717 coding sequences, as well as 24 rRNA genes and 81 tRNA genes.

### Data availability.

The DDBJ/EMBL/GenBank accession number for the whole-genome sequence of Bacillus altitudinis strain NC3 is AP025264. Raw sequencing data were deposited in the SRA under accession number DRS209021.

## References

[1] Shivaji S, Chaturvedi P, Suresh K, Reddy GSN, Dutt CBS, Wainwright M, Narlikar JV, Bhargava PM. 2006. *Bacillus aerius* sp. nov., *Bacillus aerophilus* sp. nov., *Bacillus stratosphericus* sp. nov. and *Bacillus altitudinis* sp. nov., isolated from cryogenic tubes used for collecting air samples from high altitudes. Int J Syst Evol Microbiol 56:1465–1473. doi:10.1099/ijs.0.64029-0. 16825614

[2] Fujinami S, Sato T, Ito M. 2011. The relationship between a coiled morphology and Mbl in alkaliphilic *Bacillus halodurans* C-125 at neutral pH values. Extremophiles 15:587–596. doi:10.1007/s00792-011-0389-9. 21786127

[3] Wick RR, Judd LM, Holt KE. 2019. Performance of neural network basecalling tools for Oxford Nanopore sequencing. Genome Biol 20:129. doi:10.1186/s13059-019-1727-y. 31234903 PMC6591954

[4] Wick RR. 2018. Porechop. https://github.com/rrwick/Porechop.

[5] Wick RR. 2018. Filtlong. https://github.com/rrwick/Filtlong.

[6] Martin M. 2011. Cutadapt removes adapter sequences from high-throughput sequencing reads. EMBnet J 17:10–12. doi:10.14806/ej.17.1.200.

[7] Shen W, Le S, Li Y, Hu F. 2016. SeqKit: a cross-platform and ultrafast toolkit for FASTA/Q file manipulation. PLoS One 11:e0163962. doi:10.1371/journal.pone.0163962. 27706213 PMC5051824

[8] Joshi NA, Fass JN. 2011. Sickle: a sliding-window, adaptive, quality-based trimming tool for FastQ files (version 1.33). https://github.com/najoshi/sickle.

[9] Wick RR, Judd LM, Gorrie CL, Holt KE. 2017. Unicycler: resolving bacterial genome assemblies from short and long sequencing reads. PLoS Comput Biol 13:e1005595. doi:10.1371/journal.pcbi.1005595. 28594827 PMC5481147

[10] Wick RR, Schultz MB, Zobel J, Holt KE. 2015. Bandage: interactive visualization of de novo genome assemblies. Bioinformatics 31:3350–3352. doi:10.1093/bioinformatics/btv383. 26099265 PMC4595904

[11] Parks DH, Imelfort M, Skennerton CT, Hugenholtz P, Tyson GW. 2015. CheckM: assessing the quality of microbial genomes recovered from isolates, single cells, and metagenomes. Genome Res 25:1043–1055. doi:10.1101/gr.186072.114. 25977477 PMC4484387

[12] Tanizawa Y, Fujisawa T, Nakamura Y. 2018. DFAST: a flexible prokaryotic genome annotation pipeline for faster genome publication. Bioinformatics 34:1037–1039. doi:10.1093/bioinformatics/btx713. 29106469 PMC5860143

